# Sweets and other sugary foods – a scoping review for Nordic Nutrition Recommendations 2023

**DOI:** 10.29219/fnr.v68.10488

**Published:** 2024-12-30

**Authors:** Henna Vepsäläinen, Emily Sonestedt

**Affiliations:** 1 Department of Food and Nutrition, University of Helsinki, Helsinki, Finland; 2 Nutritional Epidemiology, Department of Clinical Sciences, Lund University, Malmö, Sweden

**Keywords:** sweets, confectionary, chocolate, desserts, sugars, dietary recommendations

## Abstract

**Background:**

Sweets, chocolate, and sweet bakery products are generally high in energy and added sugar, whereas the levels of essential nutrients and fibre are low. According to sales statistics, the consumption of sweets and chocolate is high in the Nordic and Baltic countries.

**Objective:**

This scoping review describes the totality of evidence for the role of sweets and other sugary foods for health-related outcomes as a basis for setting and updating food-based dietary guidelines in the Nordic Nutrition Recommendations 2023 (NNR2023) project.

**Design:**

We conducted a literature search to identify systematic reviews published between 2011 and 2021. The literature search resulted in 756 papers, of which 12 were included in this scoping review as sources of evidence. We also used evidence from the European Food Safety Authority’s (EFSA) scientific opinion on tolerable upper intake level for dietary sugars published in 2022.

**Results:**

Most of the papers included from the search focused on chocolate or cocoa, which are rich in flavonoids. We found some evidence linking chocolate consumption with lower blood pressure, lower risk of type 2 diabetes, and improved insulin markers, but the evidence was ranked low or very low. The search did not identify systematic reviews investigating the associations between other sugary food consumption and health outcomes. In the EFSA review, conclusions were not drawn for other sugar sources than sugar-sweetened beverages. However, for fasting glucose, total cholesterol, high-density lipoprotein (HDL) cholesterol, fasting triglycerides, and uric acid, there was a statistically significant effect of high sugar intake from solid foods compared to low sugar intake.

**Conclusion:**

Because sweets, chocolate, and other sugary foods are high in energy and added sugar, and low in essential nutrients and fibre, it is reasonable to limit their consumption, which is reported high in the Nordic countries.

## Popular scientific summary

- Sweets and other sugary foods have a poor nutritional value and are usually high in energy and added sugars.
- Consumption of sweets and chocolate is high in the Nordic and Baltic countries.
- Chocolate or cocoa products high in flavanols have been associated with lower blood pressure, low-density lipoprotein (LDL) cholesterol, and markers of insulin resistance, but the quality of the evidence is low.
- Despite limited research on health outcomes, high consumption of sweets and sugary foods is unlikely to be beneficial.

Added sugar can be found in both foods and beverages. Sweets (i.e. candies), chocolate, and other confections are made primarily of sugar and are frequently consumed in the Nordic countries. Sweet bakery products, such as pastries, cakes, and cookies where flour is a main ingredient, are also rich in sugar. There are also other sugary foods, for example, ice cream. Sweets and sweet bakery products are generally high in energy, added sugar and refined grains and low in micronutrients, fibre, and proteins. The fat content varies from very low in sweets to rather high in chocolate and sweet bakery products (Table 1). Due to the reduced amount of trans fat in industrially produced foods, following their well-known association to chronic diseases, the amount of trans fat is nowadays generally low in confectioneries but can still be found in some cookies and biscuits (1). For some of the sugary foods, that is, dark chocolate, some benefits might be expected. This is because chocolate is prepared from cocoa beans, which contain high amounts of flavonoids, such as epicatechin, catechin, procyanidins, anthocyanins, and flavanols, and dark chocolate contains high levels of cocoa (2). Cocoa also contains relatively high amounts of iron, zinc, magnesium, phosphorus, and potassium (3). There are different types of chocolate with varying amount of cocoa content. The content of flavanols and procyanidins is estimated to be about 170 mg/100 g for dark chocolate and 70 mg/100 g for milk chocolate (4). In Nordic Nutrition Recommendations (NNR) 2012, the recommendation was to limit the intake of added sugars below 10 percentage of energy intake for adults and children above 6 months of age to ensure adequate intakes of micronutrients and dietary fibre (nutrient density) as well as to support a healthy dietary pattern (5). Even though it can be questioned if cocoa flavonoid products should be defined as confectioneries, the present paper gives them a certain focus as they have recently been studied substantially. The aim of this scoping review is to describe the totality of evidence for the role of sweets and other sugary foods for health-related outcomes as a basis for setting and updating food based dietary guidelines in the NNR2023 project (Box 1).

**Table 1:** Macronutrient composition of selected sugary and bakers’ confectionaries (per 100 g)

	Energy (kcal/kJ)	Fat (g)	Protein (g)	Carbohydrates (g)	Sugars (g)	Fibre (g)
Jelly candy	352/1,474	0	7.3	79.4	62.1	0
Foam candy (e.g. marshmallows)	335/1,401	0	2	80.4	67	0
Milk chocolate	564/2,358	35.2	9.2	52.9	48.5	0
Chocolate with ≥70% cacao	572/2,394	37.0	9.7	50.7	22.1	0
Danish pastries	456/1,908	27.3	6.4	45.4	13.3	2.2
Cookies	492/2,058	21.4	4.7	68.8	31.5	2.1

Information from the Swedish food database (3).

Box 1 Background papers for Nordic Nutrition Recommendations 2023

- This paper is one of many scoping reviews commissioned as part of the Nordic Nutrition Recommendations 2023 (NNR2023) project (6).
- The papers are included in the extended NNR2023 report but, for transparency, these scoping reviews are also published in Food & Nutrition Research.
- The scoping reviews have been peer reviewed by independent experts in the research field according to the standard procedures of the journal.
- The scoping reviews have also been subjected to public consultations (see report to be published by the NNR2023 project).
- The NNR2023 committee has served as the editorial board.
- While these papers are a main fundament, the NNR2023 committee has the sole responsibility for setting dietary reference values in the NNR2023 project.

## Methods

The search protocol followed the protocol developed within the NNR2023 (6) The sources of evidence used in the scoping review follow the eligibility criteria described in a previously published paper (7). No *de novo* NNR2023 systematic reviews were available for inclusion as a source of evidence in the review (8). We used evidence from the former published NNR and one NNR2023-qualified systematic review: EFSA 2022 (9) that have shown the health effects of added sugar in randomized controlled trials separately according to sugar source (beverages, solid foods, or a mixture of beverages and solid foods). In addition, we conducted a literature search for systematic reviews published since 2011. We aimed to search for papers with the consumption of sweets/candies, chocolate, sweet pastries (confectionaries), or ice cream as exposures. The search was conducted in PubMed on July 2nd 2021, and the search string used was:

(sugary[Title/Abstract] OR candy[Title/Abstract] OR sweets[Title/Abstract] OR chocolate[Title/Abstract] OR sugar-sweetened[Title/Abstract] OR confection*[Title/Abstract] OR biscuit*[Title/Abstract] OR cookie*[Title/Abstract] OR pastr*[Title/Abstract] OR bakery[Title/Abstract] OR cake*[Title/Abstract] OR “ice cream”[Title/Abstract]) AND (“2011”[Date – Publication]: “3000”[Date – Publication]) AND Humans[Filter] AND Review [Publication Type]

The search resulted in 756 papers, of which 704 were excluded based on their abstracts. The remaining 52 papers were inspected by the two authors together. To be included in the scoping review, the papers had to be systematic reviews and report the associations between sweets, confectionaries, or other sugary food consumption and a health outcome (namely, obesity, cardiovascular diseases, hypertension, type 2 diabetes, cancer, osteoporosis, mental health, other health outcomes, and total mortality). Based on these criteria, 12 papers were included in this scoping review as sources of evidence.

## Diet intake in Nordic and Baltic countries

According to sales statistics estimated by the confectionary food industry, Sweden, Denmark, and Finland have the highest consumption of sugar confectionary in the European Union (7.8, 6.3, and 5.8 kg/capita in 2018). Estonia had 3rd highest consumption of chocolate confectionary in the European Union (8.2 kg/capita in 2018), with slightly lower consumption in Finland (7.1 kg/capita), Sweden (6.8 kg/capita), Denmark (5.9 kg/capita), and Lithuania (5.0 kg/capita) (10). Even though sales data cannot accurately assess individual-level consumption, it has been shown that a moderate correlation exists between grocery purchases and food consumption for both sweets and chocolate (11). Estimated intakes of sweets and sweet bakery products assessed from the national dietary surveys are slightly lower (12). Mean intake of sweets vary between 10 g/day in Swedish men to 48 g/day in Danish women, and intake of cakes and biscuits range from 33 g/day in Finnish men to 52 g/day in Icelandic men (Table 2). In addition, mean intake of ice cream was estimated to be approximately 10 g/day in Finland and Sweden (13, 14). Table 3 shows the contribution of sugars and confectionery and fine bakery wares to added sugar intake in different age groups (9). For example, sugar and confectionery was estimated to contribute to approximately half of the added sugar intake in most of the countries (9). Sugar sweetened beverages contribute with approximately 30% of sugar intake among adolescents and adults.

**Table 2:** Intake of sweets and sweet bakery products g/day in the Nordic and Baltic countries

Country	Year of data collection (age of participants)	Men (n)	Women (n)	Sweets (g/d)	Cakes and biscuits (g/d)
Denmark	2011 (18–75 years)	1,464	1,552	38 (35)	35 (28)
				48 (51)	42 (41)
Finland	2017 (18–74 years)	780	875	12	35
				14	33
Iceland	2010 (18–80 years)	632	680	17 (30)	52 (70)
				17 (25)	43 (54)
Norway	2010 (18–70 years)	862	925	18 (25)^2^	36 (61)
				18 (23)^2^	34 (48)
Sweden	2010 (18–80 years)	792	1,005	10 (19)	33 (49)
				13 (22)	39 (33)
Estonia	2014 (18–74 years)	907	1,806	44 (38)^1^	46 (59)
				41 (50)^1^	43 (71)
Latvia	2020 (19–64 years)	470	541	32^1^	
				26^1^	
Lithuania	2019 (19–75 years)	1,348	1,562	24 (40)	
				20 (36)	

Data are provided as mean (SD). Data are retrieved from the respective country’s national dietary survey in adults and summarized in Warensjö Lemming et al. (12).

1 Sugar, sweet snacks (incl honey, candy, chocolate, jam, milk desserts, ice-cream, etc);

2 Sugar and sweets.

**Table 3:** Percent contribution of different foods and beverages to added sugar intake in different age groups and countries

Age group	Country	Sugars and confectionery^1^	Fine bakery wares^2^	Milk and dairy^3^	SSB^4^	Other^5^
Infants	Denmark	56	4	34	1	5
	Estonia	15	4	16	1	64
	Finland	82	0	0	0	18
	Latvia	39	14	18	6	23
Toddlers	Denmark	49	3	27	9	12
	Estonia	38	7	22	13	20
	Finland	61	1	18	0	20
	Latvia	36	16	21	4	23
Children	Denmark	42	1	14	33	10
	Estonia	44	7	16	14	19
	Finland	62	0	20	6	12
	Latvia	41	20	9	10	20
	Sweden	20	15	21	29	15
Adolescents 10–14 years	Denmark	41	1	14	36	8
	Estonia	44	10	11	18	16
	Finland	56	0	16	20	8
	Latvia	41	22	9	11	17
	Sweden	22	13	19	33	13
Adolescents 14–18 years	Denmark	39	1	15	37	8
	Estonia	49	8	12	17	14
	Finland	59	0	15	16	10
	Latvia	44	23	8	10	15
Adults	Denmark	45	2	10	33	10
	Estonia	54	9	11	11	15
	Finland	37	17	20	9	17
	Latvia	48	25	5	9	13
	Sweden	23	18	12	26	21
Older adults	Denmark	55	2	10	14	19
	Estonia	60	9	7	5	19
	Finland	34	25	16	3	22
	Latvia	44	27	7	7	15
	Sweden	17	29	10	12	32

Data were obtained from the EFSA report, supplementary information (Annex D) that has used data from the respective country’s national dietary surveys (30).

1 Sugar, confectionery (e.g. sweets, chocolate), and water-based desserts (e.g. sherbet);

2 Cakes, biscuits, pastries etc;

3 Sweetened yoghurt and milk-based ice cream;

4 Sugar-sweetened soft drinks and fruit drinks (including fruit and vegetable juices with added sugar);

5 Cereals, processed fruits, alcoholic beverages, and other foods with added sugars.

Comparing the intake of sweets and other sugary foods between countries is difficult as studies use different definitions of included foods. In addition, national food surveys use self-reported data that suffer from misreporting and different diet assessment methods have been used. However, it seems that sugary foods, such as sweets and sweet bakery products, are a considerable source of added sugar.

## Sweets and confectionaries and health outcomes

Of the included papers from the literature search, most focused on the consumption of chocolate (15–19), chocolate and cocoa (20–22), flavanol and flavanol-rich products (23, 24), or a combination of these (25). Two papers had ice cream and sherbet consumption as the exposures (19, 26). We found no systematic reviews estimating the associations between the consumption of biscuits, cookies, cakes, other sweet pastries, or sweets/candies and health outcomes.

### Chocolate

Due to its flavonoid content, chocolate has been suggested to be beneficial for cardiovascular health. In an umbrella review of 10 systematic reviews including both observational and intervention studies, chocolate consumption was associated with reduced risk of cardiovascular disease death, acute myocardial infarction, and stroke in observational studies (16). The associations were weaker and non-significant for coronary artery disease, atrial fibrillation, and heart failure. However, the evidence was ranked as low, and there was evidence for publication bias for cardiovascular disease death and acute myocardial infarction. In another meta-analysis consisting of one cross-sectional and six cohort studies, chocolate consumption associated with reduced risk of any cardiovascular disease (pooled data from six studies) and stroke (pooled data from three studies), but no association between chocolate consumption and heart failure (pooled data from two studies) was observed (15). The included studies were of adequate quality, and there was no evidence of significant publication bias. A meta-analysis of three cohort studies found no association between chocolate consumption and arterial fibrillation (20). Heterogeneity was reported as not important (less than 40%), and no evidence of publication bias was present.

In an umbrella review of observational studies, high chocolate consumption was associated with lower risk of type 2 diabetes. The quality of evidence was ranked as moderate (19). For most of the included studies, the conduct of the meta-analyses was rated as high, and no publication bias was indicated. In another umbrella review of systematic reviews, chocolate consumption was associated with reduced risk of diabetes in observational studies (16). However, there was evidence for publication bias. A similar result was reported in an umbrella review consisting of 24 systematic reviews of cohort studies (18), but the results were based only on one systematic review, and the confidence on the findings was critically low for most of the included reviews.

Given the high energy and added sugar contents of chocolate, several studies have examined the associations between chocolate consumption and body weight. In a review including 14 intervention studies on the effects of cocoa and/or dark chocolate in obese adults, three studies reported a reduction in body weight or BMI, while two studies found an increase in body weight or BMI (22). Five studies detected no change in body weight or BMI. The scoping review included both randomized controlled trials and cross-over trials, and the intervention periods ranged from 2 to 18 weeks. The review did not assess the quality of the included studies. A meta-analysis of cross-sectional studies showed a link between higher confectionery (mostly chocolate) consumption and lower odds of overweight or obesity among children and adolescents (17). There was no strong evidence for publication bias. Given the cross-sectional design of the meta-analysis, the effects of confectionery consumption on obesity among children and adolescents cannot be judged.

As chocolate contains flavanoids, which have been suggested to inhibit cholesterol absorption and the expression of low-density lipoprotein (LDL) cholesterol (27), studies have examined the association between chocolate consumption and blood lipid levels. In a systematic review of 10 randomized intervention studies, flavanol-rich cocoa products or dark chocolate significantly reduced total and LDL cholesterol concentrations but did not alter high-density lipoprotein (HLD) cholesterol and triglycerides (24). The effect seemed stronger in participants with high cardiovascular risk and in studies with shorter duration. The included studies were of varying quality (50% were graded as high quality), and there was evidence for heterogeneity for LDL and total cholesterol but not for HDL or triglycerides. In a meta-analysis of randomized trials, a marginally significant decrease in LDL and a similarly marginally significant increase in HDL cholesterol were observed, but there was evidence for some heterogeneity (25). Additionally, fasting triglyceride concentrations were found to reduce after intervention. However, the evidence on triglycerides was based on two small studies only. The validity of the included studies varied, and none of the studies had a low risk for bias. Summing up, the possible effects of chocolate or cocoa consumption on LDL, HDL, total cholesterol, or triglyceride levels are mainly marginal and may differ according to the participants’ personal risk profile.

In a Cochrane meta-analysis of 35 trials involving 1,804 adult participants and lasting 2 to 18 weeks, a small but statistically significant decrease in blood pressure was seen for flavanol-rich cocoa products (commercially available chocolate or flavanol-rich cocoa powder) compared with controls (23). The quality of evidence was ranked as moderate. Study quality varied, and there was a small risk of publication bias. In addition, sensitivity analyses without trials in which authors were employed by industry reduced effect sizes and statistical significance. Hooper et al. reported significant reductions in diastolic but not systolic blood pressure and mean arterial pressure after chronic chocolate intake, but no significant effects were found following acute intake (25). In another review of trials, six out of 14 studies reported improvements in blood pressure (22). In these studies, the intervention periods ranged from 2 weeks to 6 months, and the intervention was typically polyphenol-rich dark chocolate. In a meta-analysis of randomized intervention studies, Hooper et al. found an acute improvement in flow-mediated dilation at 120 min with chocolate intake (25). In another meta-analysis of intervention studies, chocolate intake influenced flow-mediated dilation at 90–150 min and at 2–18 weeks although the evidence was ranked as low or very low (16).

A meta-analysis of randomized studies observed that chocolate or cocoa flavan-3-ols significantly reduced fasting serum insulin concentrations, serum insulin after glucose challenge, and insulin sensitivity index but reported no effect on fasting glucose or HbA1c (25). The authors speculated that the effect may be associated with endothelial function. In another meta-analysis of intervention studies, chocolate intake influenced insulin resistance markers, although the evidence was ranked as low or very low (16). Most studies included in a scoping review reported no changes in glucose profiles following chocolate consumption (22).

We identified only one systematic review investigating the association between chocolate or cocoa extract consumption and mental health outcomes (21). This review included randomized, cross-sectional, and prospective studies. In a prospective design, no association between chocolate consumption and mental health measured three years later was found. One of the three randomized studies included observed a positive effect of dark chocolate drink mix on mood and another one reported that milk chocolate decreased and dark choclate increased anxiety. The third study found no relationship between cocoa extract intake and depressive symptoms or anxiety. The systematic review did not assess the quality of included studies or risk of bias. In summary, no conclusions can be drawn based on the identified evidence.

### Other sugary foods

An umbrella review including systematic reviews of observational studies found an association between ice cream consumption and lower risk of type 2 diabetes (low quality of evidence), while sherbet consumption was not associated with type 2 diabetes risk (19). Similar results were reported in a dose–response meta-analysis of cohort studies: ice cream consumption associated with a reduced risk of type 2 diabetes, whereas no link between sherbet intake and type 2 diabetes risk was observed (26). The included studies had low degree of bias, but the authors noted significant heterogeneity in the studies investigating ice cream consumption and type 2 diabetes risk.

In the EFSA Scientific opinion on upper intake level for dietary sugar from 2022, conclusions for different sugar sources were only drawn for sugar-sweetnened beverages and fruit juice. However, the effect of high versus low added sugar intake in randomized clinical trials was presented stratified by sugar source (i.e. where the sugar was from beverages, from solid foods or mixed) (9). For high compared to low added sugar intake from solid foods, there was a statistically significant effect for fasting glucose, total cholesterol, HDL-cholesterol fasting triglycerides and uric acid, while there were no statistically significant effect for LDL cholesterol, systolic blood pressure, and diastolic blood pressure (9). Data from prospective cohort studies were extracted for sugar-sweetened beverages, but not for solid foods or food groups, mainly because of the heterogenous way of assessing exposure. Importantly, there is evidence for a positive relationship between intake of sugar-sweetened beverages and risk of several chronic metabolic diseases. The EFSA Panel concluded that available data do not allow the setting an upper level of intake for added sugars. Based on the risk of developing chronic metabolic diseases and on dental caries risk, the EFSA Panel considers that the intake of added sugars should be as low as possible in the context of a nutritionally adequate diet (9).

## Mechanisms

Sweets and other sugary foods are high in energy and added sugar, while the content of essential micronutrients is generally low. Overconsumption of these highly palatable, energy-rich foods may cause weight gain, which can increase the risk of metabolic diseases. However, solid sugary foods may be more satiating than liquid sugars (i.e. sugar-sweetened beverages) (27). Because these foods mainly provide energy and are poor sources of essential micronutrients, high intake of these foods contributes to a phenomena known as micronutrient dilution, where there is less room for healthy foods and essential nutrients (28). Consumption of foods with added sugar also has other detrimental health effects. For example, the relation between high consumption of sugary foods and increased risk of dental caries is well established (9). Chocolate differs from other types of sweets, as it contains cocoa, which, in turn, is rich in flavonoids (e.g. epicatechin and procyanidins). Due to the flavonoids present in chocolate, several mechanisms for the potential beneficial health effects of chocolate have been suggested, including effects on inflammation, oxidative stress, dyslipidemia, and endothelial cell dysfunction (29). For instance, the association between higher chocolate consumption and lower risk of cardiovascular diseases can potentially be explained by the antioxidant content of cocoa, although confounding cannot be ruled out. However, commercially available milk chocolates are still fairly high in energy, added sugar, and fat, and the amount of flavonoids is quite low. Thus, it is unlikely that the potential health benefits of flavonoids would exceed the unbeneficial effects of excess energy and added sugar intake.

## Food-based dietary guidelines

Some evidence from intervention studies links chocolate or other cocoa products rich in flavanols with lower blood pressure and improved markers of glycaemic control. However, the evidence was mostly graded low or very low. In addition, there seems to be some evidence linking higher chocolate consumption with lower risk of type 2 diabetes, but as there is also evidence of publication bias, no strong conclusions can be made. In addition, the evidence regarding chocolate consumption and lower risk of cardiovascular disease can only be considered as very low or low due to the small number of studies and possible publication bias. We found no evidence on the association between chocolate consumption and other health outcomes studied, nor did we find evidence on the link between sweets, candy, or pastry consumption and the health outcomes studied. On the other hand, some evidence linked sugars from solid foods with adverse effects on fasting glucose and blood lipids.

Despite the fact that our literature review found some evidence linking higher chocolate consumption with health benefits, the evidence is limited and partly of low quality. Additionally, in many of the studies, the chocolate used as an intervention was very high in flavanols and did not resemble the typical milk chocolate used as a confectionary. Additionally, there is a lack of research examining the association between sweets/candies or sweet pastry consumption and health outcomes, which makes it challenging to set food-based dietary guidelines. However, as the aforementioned food items mainly consist of added sugar and refined grains and only include incremental amounts of essential nutrients and fibre, high consumption of these food groups is more likely to be unbeneficial. The EFSA Panel considers that the intake of added sugars should be as low as possible (9). Although the evidence for a detrimental effect on health is stronger for sugar-sweetened beverages, it is reasonable to also limit other sugary foods, especially since the majority of added sugar in the Nordic and Baltic countries is obtained from varying sorts of sugary foods followed by sugar-sweet-ened beverages.

Most of the identified systematic reviews had chocolate, cocoa, or flavanol consumption as exposures. Only a few of the systematic reviews included other food groups, such as ice cream. No systematic reviews investigating the association between sweets or sweet pastry consumption and health outcomes were identified. In addition, all but one of the identified studies included adults as participants. Thus, future studies are needed to estimate the association between sweet and confectionery consumption and health in children, adolescents, and other subgroups of interest. Due to differences in food item groupings and used terminology, it is possible that not all systematic reviews examining the association between sweets and confectionery consumption and health were identified.
